# The Role of HLA in Cord Blood Transplantation

**DOI:** 10.1155/2012/485160

**Published:** 2012-10-11

**Authors:** Catherine Stavropoulos-Giokas, Amalia Dinou, Andreas Papassavas

**Affiliations:** Hellenic Cord Blood Bank, Biomedical Research Foundation Academy of Athens (BRFAA), 4 Soranou Efessiou Street, 115 27 Athens, Greece

## Abstract

In recent years, umbilical cord blood (CB), a rich source of hematopoietic stem cells (HSC), has been used successfully as an alternative HSC source to treat a variety of hematologic, immunologic, genetic, and oncologic disorders. CB has several advantages, including prompt availability of the transplant, decrease of graft versus host disease (GVHD) and better long-term immune recovery, resulting in a similar long-term survival. Studies have shown that some degree of HLA mismatches is acceptable. This review is intended to outline the main aspects of HLA matching in different settings (related, pediatric, adult, or double-unit HSCT), its effect on transplantation outcome and the role of HLA in donor selection.

## 1. Introduction

The experience of the last 20 years indicates that cord blood transplantation is a valid alternative to bone marrow (BM) and PBSC transplants. For patients suffering from malignant or nonmalignant diseases, who do not have a matched sibling donor or a matched volunteer unrelated donor, two available alternative stem cell donor sources exist: a haploidentical transplantation from a three locus mismatched family member (parents, siblings) or an unrelated cryopreserved umbilical cord blood (CB) unit from a cord blood bank [1–4]. A low rate of graft versus host disease (GVHD) in the presence of higher HLA disparity, represents the main advantage of the umbilical cord grafts, while delayed engraftment due to limited cell dose is still the major drawback [3]. Moreover, umbilical cord blood is a viable source particularly for racial and ethnic minority patients whose genetic variations are not included in unrelated volunteer donor registries [5]. 

The role of HLA mismatches in CBT remains unclear as most transplants have been selected on low resolution class I HLA typing and allelic level class II typing. In malignant diseases, HLA mismatching is partially overcome by increasing the cell dose [6]. Recent data on associations between HLA disparity and survival, support that there is a direct association between the number of donor-recipient HLA mismatches and the risk for GVHD, while the mismatching has a greater impact on absolute mortality differences in recipients with diseases with low risk of posttransplant recurrence [7]. 

The number of CB transplantations, as well as the global inventory of CB units, are growing rapidly. CB grafts, in contrast to adults unrelated donors who need 10/10 allele level matches with the patients, have a reduced risk of severe GVHD and permit a mismatched transplantation at least in one HLA locus [8, 9]. HLA matching for unrelated CBT generally focuses on three HLA loci HLA-A,-B, and -DRB1. In order to overcome limitation in cell dose, many centers perform double unit CBT (dCBT) [10]. 

This paper focuses on the impact of HLA-matching in CBT in different settings: related, unrelated, pediatric, adult, and double CBT; the eventual inclusion of other HLA loci in the unit selection process and the future need for high resolution typing in CBT.

## 2. The MHC (Major Histocompatibility Complex)

Tissue compatibility is determined by the major histocompatibility complex (MHC), also known as the HLA system in humans, a cluster of genes located on the short arm of chromosome 6, extending about 3.6 Mb, that play a fundamental role in the acceptance and rejection of transplanted tissues [11]. The MHC is the most gene-dense region of the human genome and encompasses almost 300 genes and pseudogenes, situated in three regions called the class I, class II and class III regions. About 20% of the proteins coded by the MHC have immune-related functions [12]. Immune responses against HLA incompatibility represent a major barrier to hematopoietic stem cell transplantation (HSCT) [13, 14]. 

The class I region encodes the classical HLA molecules HLA-A, -B and -C, the nonclassical HLA-E, -F, -G, and class I-like molecules MICA and MICB. The class II region comprises the HLA-DR region (containing the DRA, DRB1, and depending on the haplotype DRB3, DRB4, or DRB5 genes), the HLA-DP region (containing the DPA1, DPB1 genes), the HLA-DQ region (containing the DQA1, DQB1 genes), as well as genes encoding proteins involved in antigen presentation. The class III region comprises genes coding for the complement cascade, cytokines, tumor necrosis factor, lymphotoxins, and heat shock proteins [11].

HLA molecules are expressed on the surface of antigen-presenting cells, displaying peptide antigens for recognition by T-cell receptors. T-cell receptors recognize antigens only if presented in the form of peptides bound to self MHC molecules, a concept known as MHC restricted recognition.

Class I molecules are constitutively expressed at varying levels on most nucleated cells and platelets. They consist of a polymorphic transmembrane **α**-chain (encoded by the corresponding MHC gene) which is associated with and stabilized by a nonpolymorphic *β*2 microglobulin chain, coded by a gene located on chromosome 15. The class-II molecules are restricted to cells of the immune system and consist of two MHC-encoded transmembrane polymorphic glycoproteins, the *α* and *β* chain-the latter being the more polymorphic. The structure of HLA Class I and Class II molecules is similar, with most of the polymorphism located in the peptide binding groove. The HLA class I molecule peptide-binding groove can bind peptides that are 8–10 amino acids long whereas HLA class II molecules bind longer peptides (12–24 amino acids). CD4^+^ T cells recognize antigens presented by class II HLA molecules and CD8^+^ T cells recognize antigens presented by class I HLA molecules.

The HLA region is the most polymorphic currently known in the human genome. According to the World Health Organization Nomenclature Committee for the HLA System, at the March 2012 update [15] (http://www.ebi.ac.uk/imgt/hla/) there are 1757 HLA-A, 2338 HLA-B, and 1304 HLA-C alleles. 

The set of HLA alleles inherited from one parent is referred to as a *haplotype *and is located on one chromosome, for example, the A1-B8-DR3 or the DRB1*15:01-DQB1*06:02 haplotypes. Linkage disequilibrium (LD) a hallmark for MHC, means that certain alleles occur together with a greater frequency than would be expected by chance. This is more frequently observed between closely located loci [11]. Certain haplotypes are common in particular ethnic groups. In hematopoietic cell transplantation from an unrelated donor, the probability of identifying an HLA-matched donor is higher when the patient and donor originate from the same ethnic group [16].

Because of the great polymorphism of HLA molecules, it became clear that serologic typing techniques were completely inadequate to cover all the diversities present in the HLA system. The serology-based method (microlymphocytotoxicity) is still in use for low resolution typing in many laboratories and to clarify the absence of some null alleles [17, 18]. The use of DNA-based techniques for HLA typing moved the field forward. DNA typing methods, are based on the nucleotide sequence information of the polymorphic DNA segments, using PCR technology. A number of HLA typing methods have been developed, mainly using PCR-SSP (sequence specific primers), reverse PCR-SSOP (sequence specific oligonucleotide probes), hybridization on solid support (microbead arrays), or sequence-based typing [18]. 

Low resolution (LR) referred to as generic typing, or 2-digit typing, corresponds to the identification of broad families of alleles (e.g., A*02) and is the equivalent of serological typing (A2). Medium resolution (MR) tissue typing techniques can define specific allele groups and subtypes. High resolution (HR) or 4-digit typing discriminates the individual alleles in each serotype (e.g., A*02:01) and resolve the tissue type to allele level, with no ambiguity [15]. The use of NMDP (http://bioinformatics.nmdp.org/HLA/hla-res-idx.html) codes can be helpful in this setting. It is recommended that selection of an unrelated donor is based on these first two sets of digits and in a second level of selection to use high resolution typing [19]. 

## 3. Related Cord Blood Transplantation

Although the primary interest in CB is an alternative unrelated donor source, CB has been used in related transplants for both malignant and nonmalignant diseases [19–21], performed almost exclusively in children. In an update of the Eurocord experience, with a median followup of 41 months after related CB transplantation for children, the survival estimate was 47 ± 5% in patients with malignancies (*n* = 96), 82 ± 7% in patients with BM failure (*n* = 33), 100% in patients with hemoglobinopathies (*n* = 52), and 70 ± 15% (*n* = 10) in patients with inborn errors of metabolism or primary immunodeficiencies [22]. By matching the Eurocord and International Bone Marrow Registry (IBMTR) [23] results of CB transplantation from HLA identical sibling donors (*n* = 113; median age, 5 years) with the results of BM transplantation (BMT) from HLA identical sibling donors (*n* = 2052; median age, 8 years), it seems that despite the lower incidence of neutrophil recovery at 1 month after CBT compared to BMT (89% versus 98%, resp.), there were no differences in 3-year survival rates (64% versus 66%, resp.), whereas incidence of grade III-IV acute GVHD and probability of chronic GVHD (3 years) were lower after CBT [24].

Related CB transplantation in patients with hemoglobinopathies, offers a probability of success comparable to that offered by BMT and is associated with a lower risk of both treatment-related mortality (TRM) and chronic GVHD, as it has been reported previously [24]. Based on this, Locatelli et al. [25], recommend collection and freezing of CB units in families in which a child is affected with genetic or hematological disease. In 2003, Reed et al. [26, 27] reported on their successful banking initiative of sibling donor CB for children with hematologic disorders, despite the challenges associated with remote-site collections. The Minesotta Group reported a case of successful CB transplantation for a patient with Fanconi anemia from unaffected HLA genotype-identical sibling selected using preimplantation genetic diagnosis [28]. The practice of preimplantation selection of HLA matched siblings for transplantation has since been established [29].

*β*-thalassemia is one of the most common single-gene inherited conditions in the world, with a particularly high prevalence in Mediterranean countries, including Greece. The Hellenic Cord Blood Bank, stores CB from healthy siblings of patients with *β*-thalassemia major. In collaboration with St Sofia Children's Hospital Stem Cell Transplant Unit, eight HLA matched units were released for transplantation and were used alone or in combination with reduced volume bone marrow from the same donor; engraftment was achieved in six out of these cases and all patients survived with 7/8 patients thalassemia-free [30].

## 4. Unrelated Cord Blood Transplantation in Children

The Cord Blood Transplantation Study (COBLT) [31], has reported the clinical outcomes of unrelated donor umbilical cord blood transplantation in pediatric patients with hematologic malignancies. All 193 patients had at least a 3/6 HLA match by low-resolution HLA-A, -B, and high resolution HLA-DRB1. The overall survival at 1 year was 57.3%, and grade III/IV aGVHD and cGVHD incidence was 19.5% and 20.2%, respectively. Higher TNC dose significantly improved engraftment. Retrospective high resolution (HR) HLA typing and the subsequent multivariate analysis revealed that while the level of original HLA match had no impact on the occurrence of grade II–IV or grade III-IV aGVHD, if the pair were matched for fewer than 5/6 alleles (HR) the probability of developing grade III/IV GVHD was significantly higher. Concerning overall survival, although there seemed to be a trend for survival advantage for 6/6 matched patients for both LR and HR typing the size of the cohort does not allow to draw definitive conclusions. The authors suggest selecting CB units that are at least 4 of 6 by LR typing at class I loci and HR typing at HLA-DRB1. Another concern is that even if HR matching decreases GVHD, overall survival may not be affected because of competing contributions of GVHD and graft-versus-Leukemia. Further analysis of larger series will provide more conclusive results regarding the impact of HLA matching on CBT.

On the other hand, for patients with non-malignant diseases the use of unrelated CB from HLA-mismatched unrelated donor will require a larger study, regarding engraftment, survival and GVHD.

In patients with hemoglobinopathies, the risk factors like the donor/recipient mismatching and cell dose, are probably amplified by the effect of multiple transfusion exposures, that might sensitize the recipient to donor alloantigens. 

In the case of severe Sickle cell disease (SCD), the cytokine milieu of SCD, which activates the inflammation and the immune activation might also promote a host-versus-graft reaction and interfere with engraftment even after myeloablative preparation [32]. In a recent phase II (BMT CTN) study of the toxicity and efficacy of unrelated donor HSCT in children with SCD, using a reduced-intensity condition regimen, one patient had 6/6 HLA antigen matching with his donor (using low-intermediate resolution typing for HLA-A-B and high resolution for HLA-DRB1), while seven patients had 5/6 HLA antigen mismatching. The median post-thaw infused CD34^+^ cell dose was 1.5*10^5^/kg. All patients achieved neutrophil recovery in median 22 days. Two patients developed grade II acute GVHD, one of these chronic GVHD and died 14 months postransplantation. According to the data a number of modifications should be done to improve the rate of engraftment after CB transplantation for severe SCD [32]. 

The use of CB from unrelated donors in *β*-thalassemia patients resulted in 77% survival in a study of 36 cases [33]. In another study investigating the feasibility of using CBT from unrelated HLA mismatched donor in 5 children with *β*-thalassemia major, all patients showed grade II or III acute GVHD and none developed extensive chronic GVHD. All patients were alive at a median followup of 303 days after transplantation with complete donor chimerism and transfusion independence [34]. 

There is a limited experience of CB transplantation in pediatric cases with idiopathic severe aplastic anemia (SAA). Information has mostly been included in registry data with very few details available [35, 36]. In a study from the Children's Hospital in San Antonio, nine children with SAA were transplanted with CB units selected from various CB banks of the USA and the choice was based on the best HLA compatibility, with at least four out of six loci matching. HLA-DRB1 compatibility between the donor and the recipient was in complete priority. At a median followup of 34 months, seven patients are alive and transfusion independent [37]. A simultaneous infusion of CD34^+^ haploidentical cells seems to improve CBT outcome for patients with SAA [38].

In pediatric patients with severe SCID there is a big discussion about the use either of mismatched related stem cells or unrelated cord blood for transplantation. According to a retrospective study on behalf of Eurocord and the Inborn Errors Working Party of the European Group for Blood and Marrow Transplantation, and although only 4 centers performed both techniques, the results did not differ significantly in terms of 5-years survival despite a higher incidence of chronic GVHD in CBT recipients [39]. CB transplantation has also been shown effective in metabolic diseases [19] in which time from diagnosis to definitive treatment may represent a crucial period to prevent further progression of the disease. The group at Duke University has reported outcomes in 20 children with Hurler syndrome who received condition regimen followed by infusion of unrelated 1, 2, or 3 HLA antigen mismatched CB. With a median followup of 905 days 17–20 children are alive with complete donor chimerism [40]. 

In a pilot study of Duke University, conducted in order to determine the safety and feasibility of intravenous administration of autologous umbilical cord blood in young children with acquired neurologic disorders, the results showed that the intravenous infusion of autologous CB is safe and feasible in young children [41].

The comparison of the results of CB and BM transplantation from unrelated donors in children is of paramount importance. It is now accepted that unrelated CB is an efficient alternative to matched unrelated BM in children and the start of a simultaneous search for BM and CB unrelated graft is supported. The final selection of unrelated donor BM versus CB should be based on the urgency of the transplant, the cell dose and HLA matching of the BM and CB unrelated donor. Moreover, CB is advantageous for children requiring urgent transplantation [9]. 

## 5. Unrelated Cord Blood Transplantation in Adults

The first unrelated cord blood transplantation was performed in 1996 and since then, more than 20.000 patients have undergone CB transplantation. In the adult setting, in a retrospective analysis of the data concerning 1525 patients with acute leukemia the results revealed that the leukemia-free survival after CBT with 4 to 6 of 6 HLA match was comparable to 7-8/8 allele-matched BMT, with grade II-IV acute and chronic GVHD and chronic GVHD lower in CBT recipients than in PBPC and BM recipients respectively. The issue of further analysis of the impact of HLA matching on transplant outcome was not addressed in this study, as for CB cell dose and not HLA matching is considered to be the limiting factor for its use: the use of a 4–6/6 match CB is considered the equivalent of a 7/8 allele matched unrelated donor when a fully matched donor is unavailable [42].

The feasibility of identifying HLA-matched donors depends on the HLA antigens of the patient and the size of the donor registries [43–45]: every patient has a mismatched donor. Intense efforts have been made to determine the “permissive” of HLA mismatches that do not increase post-transplant risks. Data for the outcomes of 1202 CB transplantations, facilitated by the New York Blood Center National Cord Blood Program, showed important differences in the small subgroups of patients with unidirectional mismatches. The graft-versus-host direction only (GVH-O) and rejection direction only (R-O) mismatches were present in 4.8% and 3.3% of the cases, respectively. According to their results, recipients of transplantation with GVH-O mismatches had neutrophil and platelet engraftment rates that were comparable to those of recipients of transplantations matched in HLA-A, -B, and -DRB1. With GVH-O mismatches, the time to engraftment was significantly faster than transplantation with R-O mismatches. In addition, patients with hematologic malignancies given GVH-O grafts had lower transplantation mortality and treatment failure compared to those with matched CB grafts [46, 47]. The practical implication is that including HLA mismatch direction in search procedures permits easy identification of grafts with unidirectional mismatches, allowing to give priority to GVH-O and to avoid R-O grafts [47]. 

Since the identification of HLA-C as a classical transplantation antigen [48], donor mismatching for HLA-C has been shown to be a risk factor after myeloablative, nonmyeloablative, unrelated donor, cord blood, marrow, and peripheral blood stem cell transplantation. A retrospective study for the effect of donor-recipient HLA matching at HLA-A, -B, -C, and -DRB1 on outcomes after CBT for leukemia and myelodysplastic syndrome, underlines the importance of HLA-C matching in CB transplantation [49]. Several reports on the association between HLA matching and survival after adult unrelated donor transplantation, showed higher transplant related mortality for transplantation HLA-A, -B, and -DRB1 matched and HLA-C mismatched, or mismatched at a single HLA-A, -B, or -DRB1 locus and mismatched at HLA-C, and transplantations mismatched at a single HLA-A, -B, or -C locus and mismatched at DRB1 [50, 51]. HLA-C is an important model for understanding differential risks conferred by allele and antigen mismatches [52]. Donor recipient pairs mismatched at HLA-C are likely to be mismatched at HLA-B because of the high degree of linkage disequilibrium between these loci [53]. Studies of prognostic factors with larger series of adults given a CB transplant are still missing and any attempt to explain the different outcomes among these series is premature. 

There is data analyzing the impact of administering a CB unit that shares a non inherited maternal HLA antigen (NIMA) with a mismatched HLA antigen in the recipient, for patients with hematologic malignancies treated with CB transplantation [54]. These noninherited maternal antigens may define “permissive” HLA mismatches and could be used to extend the genotypes of suitable matches for particular donors or CB units. Rocha et al., demonstrated that CB transplants matched for NIMA were associated with lower transplant related mortality and decreased relapse. A study by the CIBMTR, NMDP and Eurocord [55] found that NIMA matched CB transplantation resulted in superior survival and disease-free survival compared to equivalent NIMA mismatched transplantation. At the present time the role of NIMA matching in the engraftment in CB transplantation is not very clear and requires additional investigation.

From previous studies ABO incompatibility is not considered as a barrier to successful allogeneic HSCT, even though it can be associated with several immunohematologic complications, like delayed red blood cell engraftment, red cell aplasia, or hemolytic anemia. However, red blood cell alloimmunization was recently reported as an independent predictor of HLA alloimmunization [56, 57]. 

A retrospective analysis of pretransplantation sera from unrelated donor HCT recipients, showed that the presence of donor-directed, HLA specific alloantibodies was significantly associated with graft failure [58]. A recent analysis of sera from 386 myeloablative CB transplant recipients showed that the presence of donor-specific antibodies (DSA) correlated with significantly lower neutrophil recovery compared with those who lacked alloantibodies [59]. The presence of preformed DSA in double CB transplantation is predictive of higher graft failure rates and high incidence of mortality [60]. Until recently, in vitro crossmatching was used to determine compatibility between donors and recipients, and the relationship between a positive crossmatch and graft rejection in allogeneic transplantation is well established. There is strong evidence, that there is a relationship between the presence of preformed DSA and a positive crossmatch, therefore units that elicit an intense antibody response should be avoided [60, 61].

There is currently little clinical evidence suggesting an important clinical impact for HLA-DR-DQ or DP matching for CB transplantation as well as other non HLA loci like Minor Histocompatibility antigens, Killer immunoglobulin-like receptors (KIR), cytokines, chemokines, and immune response genes. 

## 6. Double CB Transplantation

In order to overcome cell dose limitations, improve engraftment rates, and immune reconstitution, a strategy consisting of administering two partially matched CB grafts called double CBT (dCBT) has been implemented. The University of Minessota program [62], a pioneer of double or sequential CBT using a nonmyeloblative regimen, has published impressive results. Although dCBT (like single CBT) shows delayed engraftment compared to other donor sources, the higher TRM is counterbalanced by lower relapse rate.

Avery et al. [63] examined the effect of HLA match on engraftment after dCBT. In almost all dCBT outcomes, single-unit dominance is observed. No relationship was found between CB/recipient match and unit dominance, even at the allelic (HR) level: a better HLA matched unit at high resolution was not more likely to become the dominant unit. Donor engraftment, is not influenced by the level of match (either at antigen or allelic level) between the two units administered; although high unit-unit match is associated with elevated initial engraftment it has no bearing to eventual graft failure. The authors recommend infusing two units with a cell dose in each unit adequate for engraftment, and 4/6 to 6/6 HLA matching to the recipient at antigen level at class I and allelic level at DRB1.

The influence of HLA matching on engraftment as well as other transplantation outcomes after double-unit CBT, should be readdressed in the future, when a very large number of cases will be available for study. Therefore, although double unit grafts have been widely adopted as a simple strategy to augment graft cells dose in unrelated donor CB transplantation, there is still little information to guide transplant centers in the selection of the graft.

Finally, more recently, it has been observed, that the percentage of viable CD34^+^ cells after thaw can vary significantly according to the bank of origin, and poor viability units were unlikely to engraft [64]. Querol et al. [65], have similarly reported variable quality between units. This raises the possibility that part of the benefit of dCBT is that, by transplanting two units, we increase the chance that at least one good quality unit, with high engraftment potential, is infused. Given that unit quality is one of the most important considerations in CBT today, the field must determine how unit quality can be reliably measured and ensured, and how poor quality units are to be investigated and/or eliminated.

## 7. Cord Blood Unit Selection

With the number of cryopreserved CB increasing and the better understanding of the factors influencing transplant outcome (cell dose, HLA match, CD34^+^ dose, etc.), a need has arisen for better strategies regarding unit selection. Organizations like the NMDP have published guidelines and transplant centers worldwide have established their own set of criteria regarding donor selection, adapted to the transplantation protocols they use and the type of patient they cater to.

NMDP strategy [53] for cord blood unit selection indicates that all patients should receive a cell dose of >2.5 × 10^7^ NC/kg. In case of double CBT, each CB should have a cell dose of >1.5 × 10^7^ NC/kg. Moreover, the patient should receive a 4/6 or better A, B, DR HLA match. For dCBT, the units should also be 4/6 or better HLA match to each other and if units have an adequate cell dose of >2.5 × 10^7^ NC/kg, a 6/6 match is preferable to a 5/6 matched unit. A very important parameter, is to avoid HLA mismatches at loci in which patients have preformed HLA antibodies. It has also been suggested that if maternal typing is available, a CB with a NIMA-shared antigen should be preferred.

 HLA matching for unrelated cord blood transplantation generally focuses on three loci (HLA-A,-B, -DRB1). Although selection currently is done to maximize matching at the antigen-level for HLA-A and -B, and at the allele-level for -DRB1, all three loci plus HLA-C are being typed by many centers at high-resolution. In a recent retrospective analysis from NMDP/CIBMTR and Eurocord [49], transplants mismatched at HLA-C were associated with higher transplant-related mortality compared to transplants matched at HLA-C; among transplants mismatched at two loci, mismatching at HLA-C and -DRB1 was associated with the highest risk of mortality. This study suggests that extended HLA matching may yield better outcomes after cord blood transplantation, although HLA match does not predict survival nor the predominant cord [66].

Gluckman and Rocha [9] reported a higher incident of graft-versus- host-disease (GVHD) and longer platelet recovery with both Class I and Class II mismatches. The effect of HLA mismatch is most important when the cell dose is low, and transplant centers are addressing the limitations in cell dose by combining two cord blood units for transplantation. Recent studies [67] examined the relationship between cell dose and HLA match in 1061 patients undergoing cord blood transplantation. Both cell dose and HLA match were independent predictors of transplant-related mortality. Patients receiving 6/6 matched CB unit had improved outcomes, regardless of cell dose. A 4/6 matched CB with cell dose >5.0 × 10^7^ NC/kg was comparable to a 5/6 matched CB unit with cell dose 2.0–5.0 × 10^7^ NC/kg. Although no consensus has yet been reached concerning intra-unit HLA match in dCBT, current practice is to maximize matching of the two units to the recipient at the antigen-level for HLA-A and -B, and at the allele-level for DRB1 with a minimum of 4/6 match [68]. 

## 8. Current Opinion in Cord Blood Banking

Since the first human CB transplant performed in 1988, CB banks (CBB) have been established worldwide for collection and cryopreservation of CB for allogeneic hematopoietic stem cells transplant [69]. CB banking includes the following phases: (1) donor recruitment, consent, and medical evaluation of the donor; (2) CB collection; short-term storage and transportation; (3) processing, testing, cryopreservation, and storage; (4) release of CB unit to transplant center; (5) quality assurance according to FACT/NETCORD standards [27].

The Netcord Foundation (http://www.netcord.org/) is a European nonprofit cooperative network of large experienced CB banks, formally established in 1998 in order to improve the quality of the grafts. The inventory of Netcord currently has more than 300.000 cryopreserved CB units ready to use, with more than 8.624 grafts shipped.

Eurocord was established in 1995 and its principal objectives were to collect data provided by CB banks and transplant centers. Eurocord (from 1988 to October 2010), has collected feedback on 6736 transplanted CB units from transplant centers in Europe and other countries. In the USA, the National Marrow Donor Program (NMDP) has established a similar CB bank network. 

International search systems have been established in order to aid transplant centers to locate eligible CB and/or adult unrelated donors (AUD). These include the Bone Marrow Donors Worldwide (BMDW): a database with HLA data and other information pertaining to CB characteristics from registries and CBB worldwide, and the EMDIS (European Marrow Donor Information System) that is a network connecting 26 registries with both CB and AUD. The two systems are complementary and account for approximately 80% of the international transplant activity [70, 71].

From collection and processing through transplantation and followup, a CB quality assurance program establishes a series of controls, quality monitors, and mechanisms that ensure product uniformity, preventing errors, and promoting continuous process and improvements. This approach has elevated the fields of CB banking and transplantation to new issues in regard to quality and process control. The Netcord Foundation in cooperation with FACT (Foundation of Accreditation of Cellular Therapy) has developed standards [72] for CBB that have been adopted by the World Marrow Donor Association (WMDA) and other National and International transplant organizations. 

The optimal number of CB units stored in order to provide any patient with a minimum 4/6 HLA matched unit, is not really known, but should approach 9 per 10.000 inhabitants [70]. An issue that should be addressed is the HLA haplotype content of the units stored: it should not only cover the commonest haplotypes of the population covered by the CBB, but also a variety of rare haplotypes or haplotypes characteristic of ethnic minorities. Targeted recruitment directed towards minorities is one of the measures already taken by several large CBB. Another measure, might be the use of HLA as a selection criterion by CB Banks, as volume unit or prereduction nucleated cell number, in order to store units not only with the most common HLA haplotypes but also for rare ones. The target would be to have an overrepresentation of rare haplotypes compared to the more common ones, making it easier to find a reasonable match for everyone, although the practical issues would be difficult to overcome.

## 9. Conclusions 

The experience of last 20 years indicates that CBT is a valid alternative method for BM and PBSC transplants. The main advantage of UCB grafts is the low rate of GVHD in the presence of higher HLA disparity, while delayed engraftment is still a mayor disadvantage due to limited cell dose. The current consensus is that CB should be at least 4/6 HLA matching for HLA-A, -B at the antigen level, and HLA-DRB1 at the allelic level. The role of additional loci as well as the impact of each individual locus remains to be determined by international studies and extended meta-analysis of large numbers of cases.

Considering the additional increasing molecular understanding of most diseases, allogeneic stem cell transplantation is headed towards a next generation of transplantation procedures: the individual adaptation in terms of graft source, engineering, and post-transplant immune interventions depending on the type of disease and underlying genetic alterations of donors and patients. Furthermore, it will allow to combine the beneficial effects of several allogeneic transplantations strategies [73], such as the early haplo-mediated neutrophil recovery, the targeted antileukemia effect of NK cells (KIR mismatch) and T-cells after selected haplo-HSCT and the long term excellent T-cell recovery after CB transplantation, but also to predict, in case of a double CB transplantation, which unit will remain as the long-term graft. All this would provide a crucial advantage for patients in need of grafts with unique genetic features such as mutations in the CCR5-coreceptor rendering carriers resistant to certain types of HIV infection: taking advantage of such types of grafts would allow curing patients with hematological malignancies and co-infection with HIV [74, 75]. Cord Blood, with its immediate availability and the possibility of having genotypically well characterized units, is a prime candidate for these applications and in the future other biological markers influencing transplant outcome or providing an advantage to carriers could be added to the selection criteria used.

Much research is ongoing to investigate the potential use of UCB stem cells in regenerative medicine. Clonal lines of multipotent cells (called the multilineage progenitor cell, MLPC) have been established from full term UCB, which can expand and differentiate into cells representing all three germinal layers. Recently, it has been shown that human unrestricted somatic stem cells (USSC's) from umbilical cord blood represent pluripotent, neonatal, nonhematopoietic stem cells with the potential to differentiate into osteoblasts, chondroblasts, adipocytes, hematopoietic, and neural cells. The mesenchymal stem cells (MSC) derived from UCB or umbilical cord (Wharton's Jelly) with their differenciation potential and immune-modulatory properties are of interest in the field of cellular therapies and regenerative medecine. MSC mediated immunosuppression, after simultaneously MSC transfusion and HSCT, has been shown to contribute to faster engraftment [76] and can be used as anti-GVHD prophylaxis [77]. CB is also a convenient source of induced pluripotent stem cells [78].

As the potential uses of cord blood extend beyond HSCT, the notion of CB banking will have to be reinvented. Cellular therapies and regenerative medicine have different immunological considerations and HLA will have a role to play that will be different: that of providing individuals with well-suited therapies. In the years to come, the better understanding of the biology of CB derived stem cells, in conjunction with new technologies will provide additional tools for the realisation of both exciting new research and novel therapeutic applications. 
